# Novel electronic properties of monoclinic MP_4_ (M = Cr, Mo, W) compounds with or without topological nodal line

**DOI:** 10.1038/s41598-020-68349-9

**Published:** 2020-07-13

**Authors:** Muhammad Rizwan Khan, Kun Bu, Jun-Shuai Chai, Jian-Tao Wang

**Affiliations:** 1 Beijing National Laboratory for Condensed Matter Physics, Institute of Physics, Chinese Academy of Sciences, Beijing, 100190 China; 2 School of Physical Sciences, University of Chinese Academy of Sciences, Beijing, 100049 China; 3 Songshan Lake Materials Laboratory, Dongguan, 523808 Guangdong China

**Keywords:** Materials science, Physics

## Abstract

Transition metal phosphides hold novel metallic, semimetallic, and semiconducting behaviors. Here we report by *ab initio* calculations a systematical study on the structural and electronic properties of $\hbox {MP}_4$ (M = Cr, Mo, W) phosphides in monoclinic *C*2/*c* ($C_{2h}^6$) symmetry. Their dynamical stabilities have been confirmed by phonon modes calculations. Detailed analysis of the electronic band structures and density of states reveal that $\hbox {CrP}_4$ is a semiconductor with an indirect band gap of 0.47 eV in association with the *p* orbital of P atoms, while $\hbox {MoP}_4$ is a Dirac semimetal with an isolated nodal point at the $\Gamma$ point and $\hbox {WP}_4$ is a topological nodal line semimetal with a closed nodal ring inside the first Brillouin zone relative to the *d* orbital of Mo and W atoms, respectively. Comparison of the phosphides with group VB, VIB and VIIB transition metals shows a trend of change from metallic to semiconducting behavior from $\hbox {VB-MP}_4$ to VIIB-$\hbox {MP}_4$ compounds. These results provide a systematical understandings on the distinct electronic properties of these compounds.

## Introduction

Transition metal phosphides (TMPs) have been attracted considerable research interest due to their structural and compositional diversity that results in a broad range of novel electronic, magnetic and catalytic properties^1–4^. This family consists of large number of materials, having distinct crystallographic structures and morphologies because of choices of different TMs and phosphorus atoms^5^. These compounds have been studied extensively due to their outstanding physical and chemical properties such as high catalytic activity^6^, good electrical conductivity^7^, and magnetocaloric behaviors^8,9^. TMPs have been appeared as an efficient catalyst for hydrogen evolution reduction (HER)^4,10–13^. For example, nanowires of FeP and $\hbox {FeP}_2$ have been used widely for hydrogen evolution in both strong alkaline and acidic aqueous solutions^10^. CoP^11^, $\hbox {CoP}_3$^12^, and $\hbox {MoP}_2$^13^ are also reported as an excellent materials for HER and oxygen evolution reduction (OER) due to their good stability. Moreover, phosphorus rich phases have been found more effective for HER and OER, and have better stability because of the presence of a large number of negatively charge P-atom centers^14,15^. In addition to electrocatalysis process, TMPs have various potential device applications, such as usage in electrotonic components, luminescent and semiconductor devices and as an anode material in lithium-ion batteries^16–19^. Moreover, some TMPs such as TaP^20^ hold topological Weyl semimetal feature, and WP has been recently reported to have Dirac like points near the Fermi level^21^. Similarly, transition metal diphosphide compounds, like $\hbox {MoP}_2$ and $\hbox {WP}_2$, were predicated as type-II Weyl topological semimetals^22^.

Topological semimetals are not only of fundamental physical interests but also of great potential for future applications in quantum computation and spintronics^23–28^. In topological semimetals, topological non-trivial band crossing points or line (line of nodes) exist in three-dimensional (3D) Brillouin zone (BZ) protected by certain symmetries^29,30^. It can be classified into Dirac semimetal^31^, Weyl semimetal^32,33^ and nodal line semimetal (NLSM)^30,34–37^. Driac semimetals have been theoretically predicted and experimentally confirmed in several materials such as $\hbox {Cd}_3\hbox {As}_2$^31^ and $\hbox {Na}_3\hbox {Bi}$^37^. Topological Weyl semimetals have paring two-fold degenerate Weyl points with opposite distinct chiralities that may be right handed or left handed and have been realized in the materials breaking the time reversal (*T*) symmetry such as pyrochlore iridate^33^ or spatial inversion (*P*) symmetry such as TaAs family of compounds^38^. In NLSMs, the bands crossing points form continuous line rather than discrete points, generally enforced due to the band inversion mechanism^39,40^ and protected by *PT* symmetry^34^. Topological NLSMs have been found in $\hbox {CaP}_3$^41^, $\hbox {Ca}_3\hbox {P}_2$^42^ phosphides and 3D graphene network structures^43–54^, etc.

In this paper, based on ab initio calculations, we systematically investigate the transition metal phosphides $\hbox {MP}_4$ ($\hbox {M} = \hbox {Cr}$, Mo, W) for the structural stability and electronic properties. These three compounds are all in monoclinic phase with *C*2/*c* ($C_{2h}^6$) symmetry, while $\hbox {CrP}_4$ and $\hbox {MoP}_4$ have been experimentally synthesized^55^ and $\hbox {WP}_4$ is not yet reported. Their mechanical stabilities are confirmed with phonon mode analysis. Electronic band calculations show that $\hbox {CrP}_4$ is a semiconductor with an indirect band gap of 0.47 eV, $\hbox {MoP}_4$ is a topological Dirac semimetal with isolated band crossing at the $\Gamma$ point, and $\hbox {WP}_4$ is a topological nodal line semimetal with a closed nodal ring inside the first BZ. We also make a comparison of the phosphides with group VB and VIIB transition metals and a trend of change from metallic to semiconducting is observed from $\hbox {VB-MP}_4$ to VIIB-$\hbox {MP}_4$ compounds.

## Results and discussion

**Figure 1:**
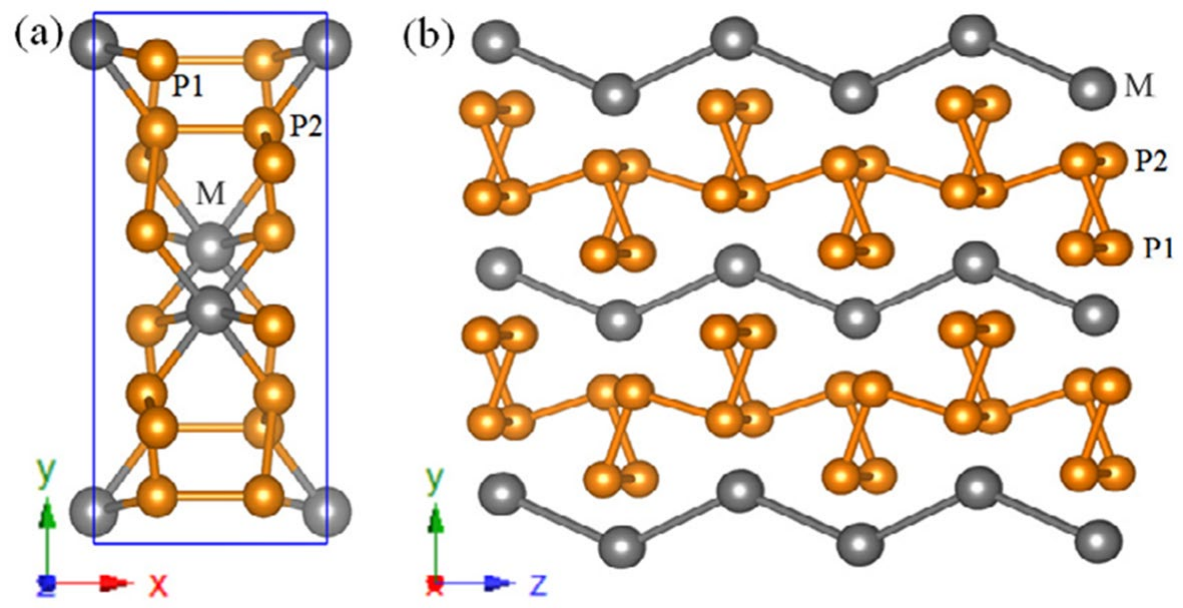
Crystal structure of $\hbox {MP}_4$ (M = Cr, Mo, W) compounds. (**a**) The unit cell in monoclinic *C*2/*c* symmetry. (**b**) the layered view. The M atoms are depicted in black while the P atoms are depicted in orange. These structures were drawn using VESTA package^76^.

We first present the structural characterization. Figure 1a shows the structure of monoclinic compounds of $\hbox {MP}_4$ ($\hbox {M} = \hbox {Cr}$, Mo, W) in *C*2/*c* ($C_{2h}^6$, No. 15) symmetry. The M atoms are depicted in black occupying the 4*e* Wyckoff positions, while there are two kinds of P atoms ($\hbox {P}_1$ and $\hbox {P}_2$) depicted in orange occupying two distinct 8*f* Wyckoff positions, respectively, as listed in Table 1. The metals environments in $\hbox {MP}_4$ compounds can be described as the octahedral coordination environment, in which metal atoms are always octahedrally surrounded by P atoms, while P atoms have tetrahedrally coordinated environment. Basically, the crystalline structure of monoclinic $\hbox {MP}_4$ compounds can be seen as a layered structure of black phosphorus in which metal atoms are inserted^56^ between two buckled phosphorus layers (Fig. 1b). Metal atoms intercalate and reorder the atomic stacks similar to Na atom insertion in black phosphorus^57^. A sandwiched structure is formed where wave like metal atom layers are in between the two buckled phosphorus layers.

**Table 1:** Atomic coordinates and Wyckoff positions for $\hbox {MP}_4$ ($\hbox {M} = \hbox {Cr}$, Mo, W) compounds in monoclinic *C*2/*c* symmetry.

Compound	Atom	Position	*x*	*y*	*z*
$\hbox {CrP}_4$	Cr	4*e*	0.0000	0.9398	0.2500
	$\hbox {P}_1$	8*f*	0.2280	0.4105	0.8238
	$\hbox {P}_2$	8*f*	0.2731	0.7815	0.1919
$\hbox {MoP}_4$	Mo	4*e*	0.0000	0.9409	0.2500
	$\hbox {P}_1$	8*f*	0.2211	0.4055	0.8168
	$\hbox {P}_2$	8*f*	0.2774	0.7779	0.1893
$\hbox {WP}_4$	W	4*e*	0.0000	0.9406	0.2500
	$\hbox {P}_1$	8*f*	0.2219	0.4056	0.8173
	$\hbox {P}_2$	8*f*	0.2768	0.7780	0.1884

There are three unique types of bonds in monoclinic compounds $\hbox {MP}_4$, namely M-$\hbox {P}_1$, M-$\hbox {P}_2$, and $\hbox {P}_1$-$\hbox {P}_2$ chemical bonds. In $\hbox {CrP}_4$, the bond lengths are 2.277–2.373 Å for Cr-$\hbox {P}_1$, 2.316 Å for Cr-$\hbox {P}_2$, and 2.215–2.240 Å for $\hbox {P}_1$-$\hbox {P}_2$; in $\hbox {MoP}_4$, the bond lengths are 2.396–2.456 Å for Mo-$\hbox {P}_1$, 2.456 Å for Mo-$\hbox {P}_2$, and 2.208–2.243 Å for $\hbox {P}_1$-$\hbox {P}_2$; while in $\hbox {WP}_4$, the bond lengths are 2.398–2.477 Å for W-$\hbox {P}_1$, 2.453 Å for W-$\hbox {P}_2$, and 2.215–2.245 Å for $\hbox {P}_1$-$\hbox {P}_2$. Meanwhile, there are three distinct types of bond angles depicted as $\angle \hbox {P}_1$-M-$\hbox {P}_1$, $\angle \hbox {P}_2$-M-$\hbox {P}_2$ and $\angle \hbox {P}_1$-M-$\hbox {P}_2$. For $\hbox {CrP}_4$, the bond angles are $90.03^{\circ }$ for $\angle \hbox {P}_1$-Cr-$\hbox {P}_1$, $85.30^{\circ }$ for $\angle \hbox {P}_2$-Cr-$\hbox {P}_2$, and $92.37^{\circ }$ for $\angle \hbox {P}_1$-Cr-$\hbox {P}_2$; for $\hbox {MoP}_4$, the bond angles are $88.19^{\circ }$ for $\angle \hbox {P}_1$-Mo-$\hbox {P}_1$, $83.80^{\circ }$ for $\angle \hbox {P}_2$-Mo-$\hbox {P}_2$, and $94.0^{\circ }$ for $\angle \hbox {P}_1$-Mo-$\hbox {P}_2$; while for $\hbox {WP}_4$, the bond angles are $88.19^{\circ }$ for $\angle \hbox {P}_1$-W-$\hbox {P}_1$, $84.16^{\circ }$ for $\angle \hbox {P}_2$-W-$\hbox {P}_2$, and $93.83^{\circ }$ for $\angle \hbox {P}_1$-W-$\hbox {P}_2$. It can be seen that the bond lengths between P-P atoms are almost same in the three $\hbox {MP}_4$ compounds, while the bond lengths between Mo-P and W-P atoms are clearly larger than that between Cr-P atoms. Meanwhile, $\angle \hbox {P}_1$-M-$\hbox {P}_2$ are found larger than the other angles in all $\hbox {MP}_4$ compounds. The calculated equilibrium lattice parameters, bond lengths, and bond angles for $\hbox {MP}_4$ compounds are listed in Table 2. It is seen that our calculated structural parameters matches well with the reported experimental and calculated data^55,58,59^.

**Table 2:** Calculated equilibrium lattice parameters (*a*, *b*, *c* and $\beta$), bond lengths ($d_{M-P1}$, $d_{M-P2}$, and $d_{P-P}$), and electronic band gap $E_g$ for $\hbox {MP}_4$ ($\hbox {M} = \hbox {Cr}$, Mo, W) compounds, comparing with experimental and previously calculated data^55,58,59^.

Compound	Method	*a*(Å)	*b*(Å)	*c*(Å)	$\beta$($^{\circ }$)	$\hbox {d}_{(M-P1)}$(Å)	$\hbox {d}_{(M-P2)}$(Å)	$\hbox {d}_{(P-P)}$(Å)	$\angle$P-M-P($^{\circ }$)	$\hbox {E}_g$ (eV)
$\hbox {CrP}_4$	PBE	5.196	10.754	5.717	110.42	2.277–2.373	2.316	2.215–2.240	85.30–92.37	0.47
	Exp^55^	5.191	10.760	5.771	110.65					
	PBE^58^	5.170	10.684	5.692	110.03					0.63
$\hbox {MoP}_4$	PBE	5.337	11.207	5.855	110.72	2.396–2.456	2.456	2.208–2.243	83.80–94.0	Semimetal
	Exp^55^	5.313	11.139	5.820	110.64					
	PBE^59^	5.268	11.090	5.798	110.80					
$\hbox {WP}_4$	PBE	5.344	11.195	5.876	110.95	2.398–2.475	2.453	2.215–2.245	84.16–93.83	Semimetal

**Figure 2:**
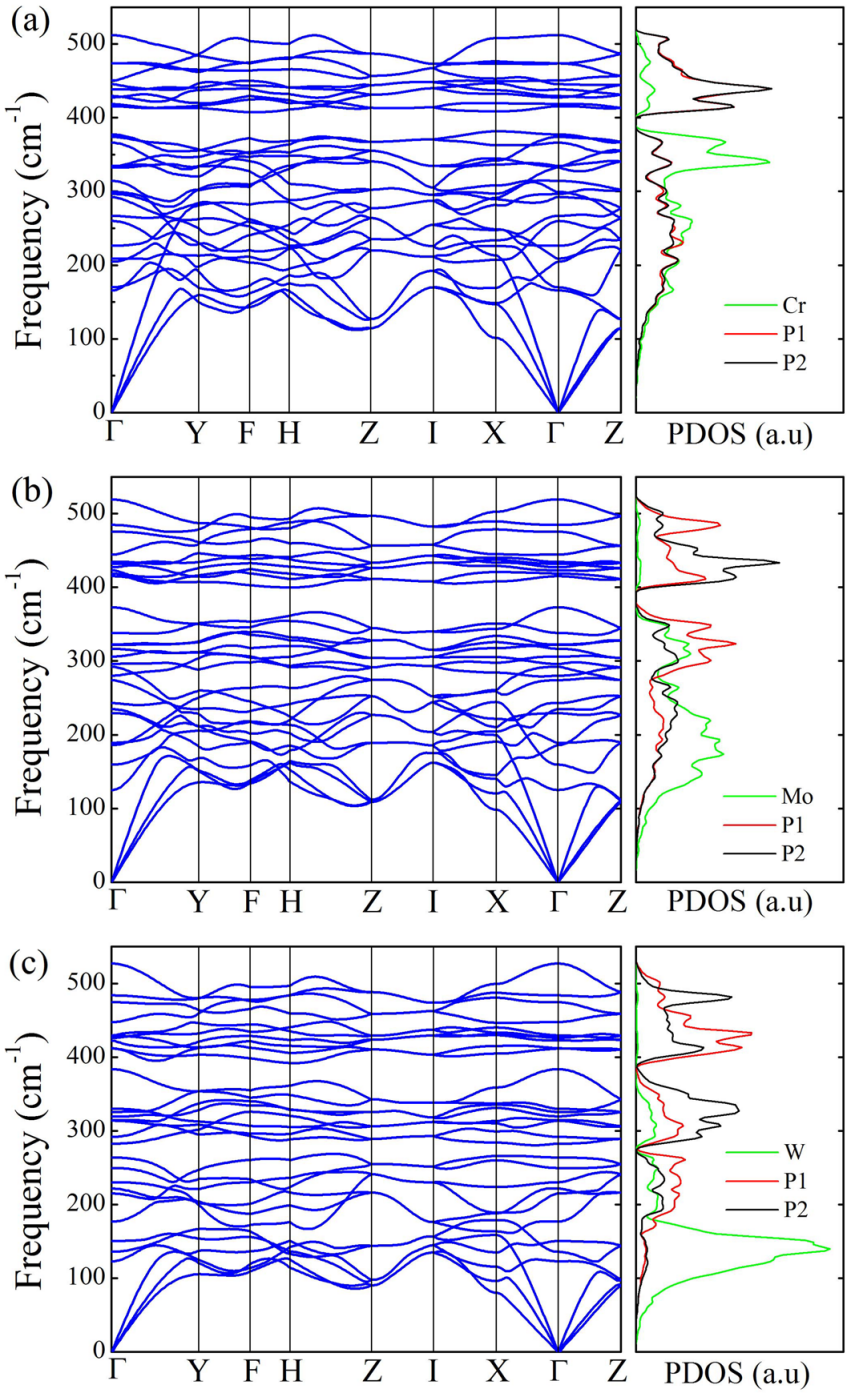
Phonon band structures and density of states (PDOS) for $\hbox {MP}_4$ (M = Cr, Mo, W) compounds at equilibrium lattice parameters. The lower frequency modes are mainly contributed by the metal atoms because of their heavier masses while the higher frequency modes are mainly contributed by the P atoms with lighter masses.

To examine the dynamical stability of $\hbox {MP}_4$ compounds, we have calculated the phonon band structures and partial phonon density of states (PDOS) with equilibrium lattice parameters in a $2\times 2\times 2$ supercell, as shown in Fig. 2. For $\hbox {CrP}_4$, $\hbox {MoP}_4$ and $\hbox {WP}_4$, no imaginary frequencies occur in the whole BZ and PDOS, thus confirming the structural stability of the three compounds. There are some similarities in the phonon band structures and PDOS for $\hbox {CrP}_4$, $\hbox {MoP}_4$ and $\hbox {WP}_4$ due to the same space symmetry groups and elementary components for the three compounds. The highest vibrational frequencies all happen near the $\Gamma$ point and the values are 519.8 cm$^{-1}$ for $\hbox {CrP}_4$, 521.8 cm$^{-1}$ for $\hbox {MoP}_4$ and 526.8 cm$^{-1}$ for $\hbox {WP}_4$, respectively. It is seen from the PDOS that the lower frequency modes are mainly contributed by the metal atoms because of their heavier masses while the higher frequency modes are mainly contributed by the P atoms with lighter masses.

**Figure 3:**
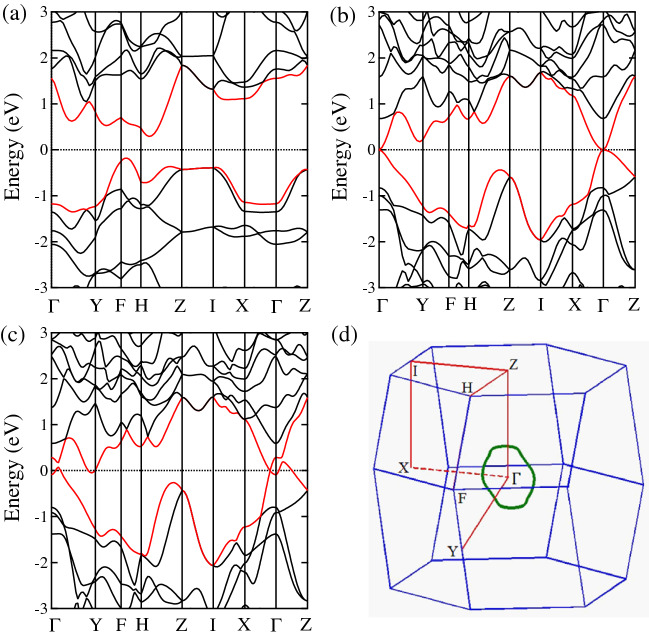
Electronic band structures for (**a**) $\hbox {CrP}_4$, (**b**) $\hbox {MoP}_4$ and (**c**) $\hbox {WP}_4$ at equilibrium lattice parameters using HSE06 functional (without spin-orbital coupling). (**d**) The BZ with several high-symmetry points indicated at $\Gamma$ (0.00, 0.00, 0.00), Y (0.3067, 0.3067, 0.0440), F (0.3631, 0.3631, 0.3937), H (0.2503, 0.2503, 0.6943), Z (0.00, 0.00, 0.50), I (0.50, $-0.50$, 0.50), and X (0.50, $-0.50$, 0.00). The nodal ring (green circle) in (**d**) is formed by band crossing points for $\hbox {WP}_4$ compound were plotted using MATLAB software.

Next we discuss the electronic properties of $\hbox {MP}_4$ ($\hbox {M} = \hbox {Cr}$, Mo, W) compounds. Figure 3 represents the calculated electronic band structures along the high symmetry directions of the BZ using HSE06 functional^60^ and the fermi energy ($E_F$) is set to zero. For $\hbox {CrP}_4$ as shown in Fig. 3a, the conduction band minimum (CBM) is located along H-Z direction and valence band maximum (VBM) is located along F-H direction, showing a semiconducting behavior with an indirect band gap of 0.47 eV, which is smaller than the reported direct band gap of 0.63 eV^58^. For $\hbox {MoP}_4$ as shown in Fig. 3b, the lowest conduction band and highest valence band are degenerate at $\Gamma$ point near the $E_F$, indicating that $\hbox {MoP}_4$ is a Dirac semimetal with a four-fold degenerate Dirac point at the $\Gamma$ point^61^. Moreover, our calculations show that the valence and conduction bands of $\hbox {WP}_4$ exhibit linear dispersion near the $E_F$ and cross along the $\Gamma$-X high symmetry direction (Fig. 3c) due to the band inversion mechanism^39,40^. To further explore the topological electronic properties, we establish a tight binding (TB) model using the maximally localized Wannier functions (MLWFs)^62,63^ to search the nodal points in the 3D BZ. We find that the nodal points (or band crossing points) of valence and conduction bands in $\hbox {WP}_4$ form a continuous nodal ring in the full BZ (see Fig. 3d), thus, $\hbox {WP}_4$ can be termed as a topological nodal line semimetal with a closed nodal ring protected by *PT* symmetry^34,35,41^.

**Figure 4:**
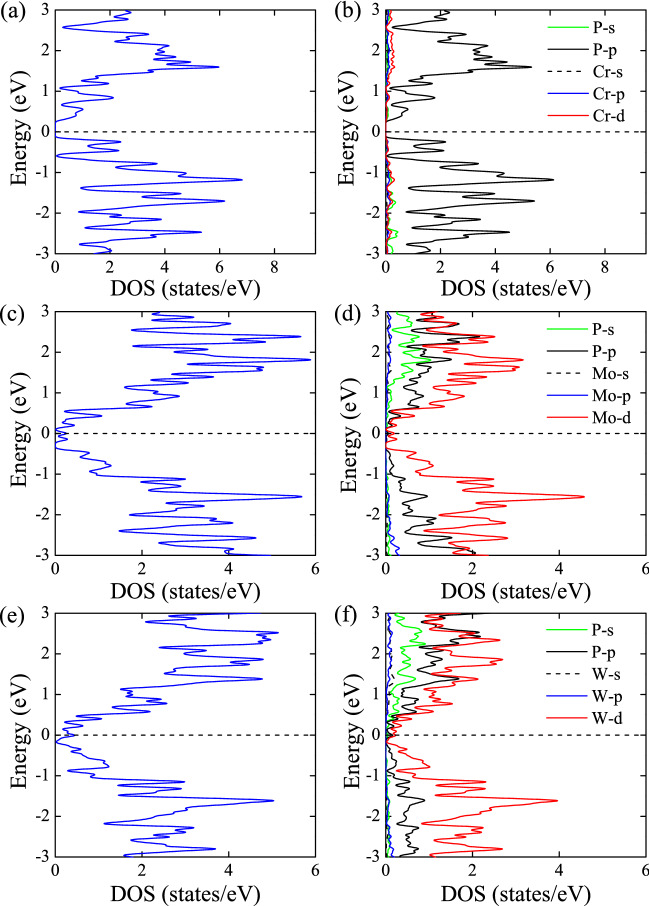
Total and partial density of states (DOS) for $\hbox {MP}_4$ (M = Cr, Mo, W) compounds at equilibrium lattice parameters using HSE06 functional (without spin-orbital coupling). (**a, b**) Total and partial DOSs for $\hbox {CrP}_4$; (**c, d**) Total and partial DOSs for $\hbox {MoP}_4$; and (**e, f**) Total and partial DOSs for $\hbox {WP}_4$.

It is interesting to notice that although Cr, Mo and W are all in the VIB group of the Periodic Table of Elements, $\hbox {CrP}_4$ is an indirect band gap semiconductor, $\hbox {MoP}_4$ is a Dirac semimetal with a single nodal point, and $\hbox {WP}_4$ is a nodal line semimetal with a closed nodal ring. The metallicity of $\hbox {CrP}_4$, $\hbox {MoP}_4$, and $\hbox {WP}_4$ grows with the increase of the elementary ordinal from **3d** to **5d** transition metals. To further understand the electronic properties, we have plotted the total and partial density of states (DOS) of $\hbox {MP}_4$ compounds as shown in Fig. 4. For $\hbox {CrP}_4$ (Fig. 4a), there is a band gap of 0.47 eV as depicted in Fig. 3a. The states around the Fermi level are mainly contributed by the *p* states of P atoms (Fig. 4b), relative to the covalent bonds between P-P atoms. For $\hbox {MoP}_4$ (Fig. 4c), there is a little peak on the Fermi level, the states at the Fermi level are mainly composed of *d* orbital of Mo atoms (see Fig. 4d). Moreover, for $\hbox {WP}_4$ (Fig. 4e), there is a little peak on the Fermi level, but larger than that in $\hbox {MoP}_4$, the states at the Fermi level are predominantly composed of P-*p* orbital and W-*d* orbital (Fig. 4f). It can be inferred that the electronic behaviors in $\hbox {CrP}_4$ are mainly dominated by the P-P covalent bonds in $\hbox {CrP}_4$, so that $\hbox {CrP}_4$ tend to be a semiconductor due to covalent bonding properties between P-P atoms. While in $\hbox {MoP}_4$ and $\hbox {WP}_4$, the electronic properties are largely determined by the metal atoms which have metallic bonds with P atoms, so that they show semimetallic properties. The small peaks on the Fermi level in $\hbox {MoP}_4$ and $\hbox {WP}_4$ semimetals are related to the band touching point between the top of valance and the bottom of conduction bands. Similar DOSs around the Fermi level are also found in $\hbox {CaP}_3$ family of nodal line semimetals^41^.

We have further examined the band structures of $\hbox {MoP}_{4}$ and $\hbox {WP}_{4}$ with spin-orbital coupling (SOC) as shown in Fig. S1 in Supplementary Information. For $\hbox {MoP}_4$, the SOC induced band gap is about 0.1 meV at the $\Gamma$ point, while for $\hbox {WP}_4$, the SOC induced band gap is about 29 meV along the high-symmetric X-$\Gamma$ direction. We can see that when SOC is included, $\hbox {MoP}_4$ and $\hbox {WP}_4$ become strong topological insulators with the symmetry-based indicators^64–66^ ($z_2$, $z_2$, $z_2$, $z_4$) as (0,0,0,1), like as the finding in $\hbox {CaP}_3$ family of materials^41^.

In order to better understand the electronic properties of VIB-$\hbox {MP}_4$ ($\hbox {M}= \hbox {Cr}$, Mo, W) compounds, we have also examined the electronic properties of the $\hbox {VP}_4$, $\hbox {NbP}_4$, $\hbox {TaP}_4$, $\hbox {MnP}_4$, $\hbox {TcP}_4$ and $\hbox {ReP}_4$, while V, Nb and Ta are in the VB group, and Mn, Tc and Re are in the VIIB group, which are all next to Cr, Mo and W in the Periodic Table of Elements. The $\hbox {TcP}_4$ and $\hbox {ReP}_4$ are experimentally synthesized by the reaction of their constituent elements^67–69^. The calculated equilibrium lattice parameters and electronic band structures are given in Table S1 and Fig. S2 in Supplementary Information, respectively. The structural parameters and electronic behavior that is, $\hbox {VP}_4$ is metallic and $\hbox {MnP}_4$ is a semiconductor reported by Gong et al.^58^. We find that $\hbox {VB-MP}_4$ ($\hbox {M}= \hbox {V}$, Nb, Ta) have metallic behavior, while VIIB-$\hbox {MP}_4$ ($\hbox {M}= \hbox {Mn}$, Tc, Re) are semiconductors. It is clearly seen that from $\hbox {VB-MP}_4$ to VIIB-$\hbox {MP}_4$, the metallicity of these phosphides grow weaker with a change from metallic to semiconducting, while from top (**3d**) to bottom (**5d**) in each group, the metallicity of these phosphides grow stronger. So it is reasonable that $\hbox {CrP}_4$ should be a semiconductor, $\hbox {MoP}_4$ is a semimetal with isolated nodal points and $\hbox {WP}_4$ is a topological nodal line semimetal with a line of nodes.

## Conclusions

In conclusion, we have performed a systematic ab initio study on $\hbox {MP}_4$ ($\hbox {M} = \hbox {Cr}$, Mo, W) monoclinic compounds. Their dynamical stabilities have been confirmed by phonon modes calculations. Electron band structures calculations show that $\hbox {CrP}_4$ is an indirect band gap semiconductor with a narrow band gap of 0.47 eV, $\hbox {MoP}_4$ is Dirac semimetal and $\hbox {WP}_4$ is considered as a new candidate for topological nodal line semimetal with a closed nodal ring in the first BZ protected by the *PT* symmetry. The electronic density of states calculations indicate that in $\hbox {CrP}_4$, the valence and conduction bands near the Fermi level are mainly contributed by the *p* orbitals of P atoms, while in $\hbox {MoP}_4$ and $\hbox {WP}_4$, there is a little peak on the Fermi level and the energy bands are mainly composed of *d* orbitals of Mo and W atoms, respectively. We also make a comparison of the phosphides with group VB and VIIB transition metals and a trend of change from metallic to semiconducting is observed from $\hbox {VB-MP}_4$ to VIIB-$\hbox {MP}_4$ compounds. These results provide a systematic understanding and pave the way for further experimental explorations on the transition metal phosphides.

## Methods

Our calculations were carried out using the density functional theory as implemented in the Vienna ab initio simulation package (VASP)^70^. The projector augmented wave (PAW)^71^ method was adopted with valence electrons of $3s^23p^3$ for P, $3p^63d^54s^1$ for Cr, $4p^64d^55s^1$ for Mo, and $5p^65d^46s^1$ for W. Generalized gradient approximation (GGA) developed by Perdew, Burke and Ernzerhof (PBE)^72^ is used as the exchange-correlation potential. A $5 \times 8 \times 6$ Monkhorst-Pack grid of BZ sampling is used and an energy cutoff of 500 eV is set for the plane-wave basis. The structures are fully optimized until the total energy difference is less then 10$^{-6}$ eV and convergence criteria for atomic forces is set to be 10$^{-3}$ eV/Å. The electronic properties are calculated with the Heyd–Scuseria–Ernzerhof hybrid functional (HSE06)^60^ and the phonon properties are calculated with phononpy package^73^. To further explore the topological electronic properties, we establish a tight binding (TB) model using the maximally localized Wannier functions (MLWFs)^62,63^ implemented in Wannier90 package^74^ and searched the band crossing points in the entire BZ with WannierTools pacakge^75^.

## Supplementary information

Supplementary information
