# Supplementary material for: Novel electronic properties of monoclinic MP4 (M = Cr, Mo, W) compounds with or without topological nodal line
Source: Sci Rep. 2020 Jul 13;10:11502. doi: 10.1038/s41598-020-68349-9 (PMC7359338; doi:10.1038/s41598-020-68349-9)
Supplement: Supplementary file 1 — Supplementary information [file 41598_2020_68349_MOESM1_ESM.docx]

**Supporting information：**

**Novel electronic properties of monoclinic MP_4_(M = Cr, Mo, W) compounds with or without topological nodal line**

Muhammad Rizwan Khan,^1,2^ Kun Bu,^1,2^ Jun-Shuai Chai,^1,2^ Jian-Tao Wang^1,2,3*^

^1^Beijing National Laboratory for Condensed Matter Physics, Institute of Physics, Chinese Academy of Sciences, Beijing 100190, China

^2^School of Physical Sciences, University of Chinese Academy of Sciences, Beijing 100049, China

^3^Songshan Lake Materials Laboratory, Dongguan, Guangdong 523808, China

*E-mail: [wjt@aphy.iphy.ac.cn](mailto:wjt@aphy.iphy.ac.cn)

1. **Electronic band structures of MoP_4_ and WP_4_ with spin-orbital coupling**

To better understand the electronic properties, we have also calculated the electronic band structures of MoP_4_ and WP_4_ with spin-orbital coupling (SOC). Figure S1a shows the band structure for MoP_4_ with SOC. When SOC is considered, the nodal point at the Γ point can be gapped and there is a small band gap about 0.1 meV as shown in Fig. S1b. Figure S1c shows the band structure for WP_4_ with SOC. The SOC induced band gap is about 29 meV along the X-Γ direction as shown in Fig. S1d. We can see that when SOC is included, the gaps open along the nodal line (or nodal points) and these materials become strong topological insulators with the symmetry-based indicators^1-3^ (z_2_, z_2_, z_2_, z_4_) as (0,0,0,1), like as the finding in CaP_3_ family of materials^4^.


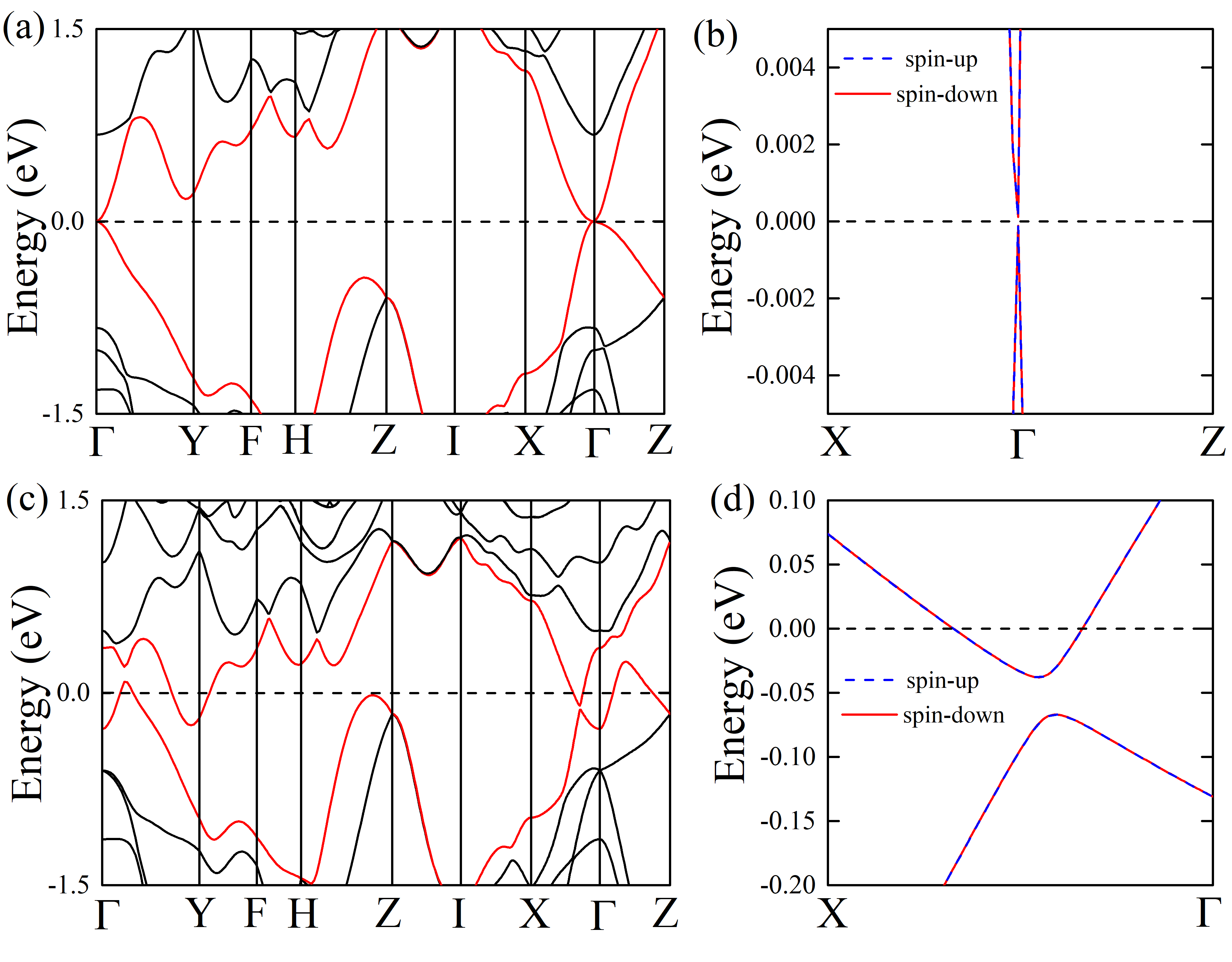


**Figure S1**: **Electronic band structures with spin-orbital coupling at equilibrium lattice parameters.**  (a) for MoP_4_, (b) for MoP_4_ along the path X-Γ-Z, (c) for WP_4_, (d) for WP_4_ along the path X-Γ.

1. **Electronic properties for VB-MP_4_ and VIIB-MP_4_ compounds**

In order to better understand the electronic properties of **VIB**-MP_4_ (M = Cr, Mo, W) monoclinic compounds, we have also examined the structural and electronic properties of the **VB**-MP_4_ (VP_4_, NbP_4_, TaP_4_) and **VIIB**-MP_4_ (MnP_4_, TcP_4_ and ReP_4_) compounds, which are all next to Cr, Mo and W in the Periodic Table of Elements. The **VB**-MP_4_ compounds such as NbP_4_ and TaP_4_ are optimized in the monoclinic crystal structure with C2/c ($C_{2h}^{6}$, No. 15) symmetry^5^. There are three exclusive types of chemical bonds in these compounds, namely M-P_1_, M-P_2_, and P_1_-P_2_ chemical bonds. In NbP_4_, the bond lengths are 2.489-2.543 Å for Nb-P_1_, 2.535 Å for Nb-P_2_, and 2.224-2.239 Å for P_1_-P_2_; in TaP_4_, the bond lengths are 2.473-2.531 Å for Ta-P_1_, 2.516 Å for Ta-P_2_, and 2.222-2.237Å for P_1_-P_2_. Meanwhile, there are three distinct types of bond angles characterized as $\angle\text{P}_{1}\text{-M-P}_{1}$, $\angle\text{P}_{2}\text{-M-P}_{2}$ and $\angle\text{P}_{1}\text{-M-P}_{2}$.

Table S 1 : Calculated equilibrium lattice parameters (a, b, c and β), bond lengths ($\text{d}_{\text{M-P}_{1}}$,$\text{d}_{\text{M-P}_{2}}$,$\text{d}_{\text{M-P}_{3}}$,$\text{d}_{\text{M-P}_{4}}$and $\text{d}_{\text{P-P}}$), and electronic band gap E_g_ for MP_4_ (M = Nb, Ta, Tc, Re) compounds of group VB and VIIB, comparing with experimental and previously calculate data.

| Compound | Method | *a*(Å) | *b(*Å*)* | *c*(Å) | *β*  (deg) | $\text{d}_{\text{M-P}_{1}}$ (Å) | $\text{d}_{\text{M-P}_{2}}$ (Å) | $\text{d}_{\text{M-P}_{3}}$ (Å) | $\text{d}_{\text{M-P}_{4}}$ (Å) | $\text{d}_{\text{P-P}}$ (Å) | $\angle\text{P-M-P}$ (deg) | E_g_ (eV) |
| --- | --- | --- | --- | --- | --- | --- | --- | --- | --- | --- | --- | --- |
| NbP_4_ | PBE | 5.413 | 11.558 | 6.01 | 110.68 | 2.489-2.543 | 2.535 |  |  | 2.224-2.239 | 82.59-95.92 | Metal |
| TaP_4_ | PBE | 5.409 | 11.486 | 6.01 | 110.83 | 2.47-2.253 | 2.516 |  |  | 2.222-2.237 | 83.15-95.01 | Metal |
| TcP_4_ | PBE | 6.271 | 9.264 | 10.897 | 90 | 2.344-2.364 | 2.396 | 2.365 | 2.430 | 2.209-2.268 | 84.55-95.89 | 0.831 |
|  | Exp ^7^ | 6.228 | 9.215 | 10.837 | 90 |  |  |  |  |  |  |  |
|  | LDA^8^ | 6.280 | 9.241 | 10.827 | 90 |  |  |  |  |  |  | 0.91 |
| ReP_4_ | Exp ^6^ | 6.267 | 9.292 | 10.932 | 90 | 2.360-2.380 | 2.406 | 2.366 | 2.435 | 2.193-2.228 | 85.30-92.37 | 0.737 |
|  | PBE | 6.23 | 9.231 | 10.854 | 90 |  |  |  |  |  |  |  |

The **VIIB**-MP_4_ such as TcP_4_ and ReP_4_ are crystallized in the orthorhombic structure with *Pbca* ($D_{2h}^{15}$, No. 61) symmetry^6-8^. In these compounds, there are five distinct types of bonds, known as M-P_1_, M-P_2_, M-P_3_, M-P_4_, and P_1_-P_2_ chemical bonds. In TcP_4_, the bond lengths are 2.344-2.364 Å for Tc-P_1_, 2.396 Å for Tc-P_2_, 2.365 Å for Tc-P_3_, 2.430 Å for Tc-P_4_, and 2.209-2.268 Å for P_1_-P_2_; in ReP_4_, the bond lengths are 2.360-2.380 Å for Re-P_1_, 2.406 Å for Ta-P_2_, 2.366 Å for Re-P_3_, 2.435 Å for Re-P_4_, and 2.193-2.228 Å for P_1_-P_2_. The calculated equilibrium lattice parameters for **VB**-MP_4_ and **VIIB**-MP_4_ compounds are listed in Table S1, comparing with available experimental and calculated data^6-8^.


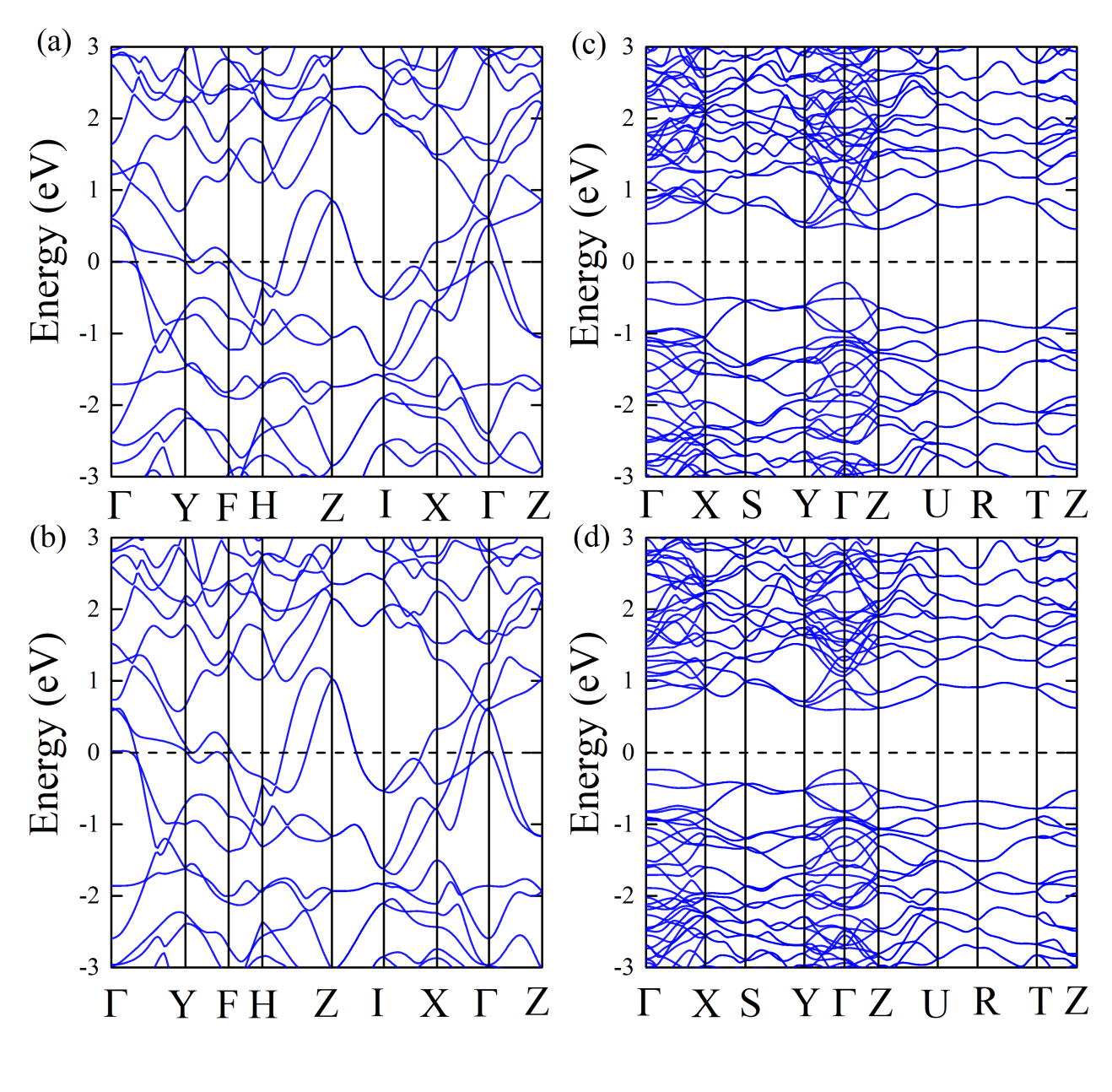


Figure S2: Electronic band structures for (a) NbP_4_, (b) TaP_4_, (c) TcP_4_ and (d) ReP_4_ at equilibrium lattice parameters using PBE-GGA functional. NbP_4_ and TaP_4_ have metallic behavior, while TcP_4_ and ReP_4_ are semiconductors.

Next, we explain the electronic properties of **VB**-MP_4_ and **VIIB**-MP_4_ compounds. For NbP_4_ (Fig. S2a) and TaP_4_ (Fig. S2b), the valence and conduction bands are overlap with each other, showing a metallic behavior. On the other hand, for TcP_4_ compound (Fig. S2c), the conduction band minimum (CBM) is located at Z point and valence band maximum (VBM) is located at Γ point, respectively, showing an indirect band gap semiconductor with gap of 0.831 eV. Meanwhile, for ReP_4_ (Fig. S2d), the CBM is located along the Γ-Y direction and VBM is at the Γ point, which also reveal an indirect band gap semiconductor with gap of 0.737 eV.

The electronic properties of **VB**-MP_4_, **VIB**-MP_4_, and **VIIB**-MP_4_ are summarized in Table S2. It is clearly seen that from **VB**-MP_4_ to **VIIB**-MP_4_, the metallicity of these phosphides grows weaker with a change from metallic to semiconducting, while from top (**3d**) to bottom (**5d**) in each group, the metallicity of these phosphides grows stronger. These results further support our finding that CrP_4_ should be a semiconductor, MoP_4_ is a semimetal with isolated nodal points and WP_4_ is a topological nodal line semimetal with line of nodes.

Table S2: The electronic properties of **VB**-P_4_, **VIB**-P_4_, and **VIIB**-P_4_ at equilibrium lattice parameters using PBE-GGA functional.

| TM | **VB-**MP_4_ | **VIB-**MP_4_ | **VIIB-**MP_4_ |
| --- | --- | --- | --- |
| **3d** | VP_4_ (metal)^5^ | CrP_4_ (semiconductor) | MnP_4_( semiconductor)^5^ |
| **4d** | NbP_4_ (metal) | MoP_4_ (semimetal) | TcP_4_ (semiconductor) |
| **5d** | TaP_4_ (metal) | WP_4_ (semimetal) | ReP_4_ (semiconductor) |

**References**

1. Po, H. C., Vishwanath, A. & Watanabe, H. Symmetry-based indicators of band topology in the 230 space groups. *Nat. Commun.* **8**, 1-9, doi:10.1038/s41467-017-00133-2 (2017).
2. Song, Z., Zhang, T., Fang Z. & Fang, C. Quantitative mappings between symmetry and topology in solids. *Nat. Commun.* **9**, 1-7, doi:10.1038/s41467-018-06010-w (2019).
3. Song, Z., Zhang, T. & Fang, C. Diagnosis for nonmagnetic topological semimetals in the absence of spin-orbital coupling. *Phys. Rev. X* **8**, 031069, doi:10.1103/PhysRevX.8.031069 (2018).
4. Xu, Q., Yu, R., Fang, Z., Dai, X. & Weng, H. Topological nodal lines semimetals in the CaP_3_ family of materials. *Phys. Rev. B* **95**, 045136, doi:10.1103/PhysRevB.95.045136 (2017).
5. Gong, N., Deng, C., Wu, L., Wan, B., Wang, Z., Li, Z. & Gao, F. Structural diversity and electronic properties of 3d transition metal tetraphosphides, TMP_4_ (TM = V, Cr, Mn, and Fe). *Inorg. Chem.* **57**, 9385-9392, doi:10.1021/acs.chemmater.6b05052 (2018).
6. Jeitschko, W. & Rühl, R. Synthesis and crystal structure of diamagnetic ReP_4_, a polyphosphide with Re pairs. *Acta. Crystallogr. B* **35**, 1953-1958, doi:10.1107/S0567740879008232 (1979).
7. Rühl, R., Jeitschko, W. & Schwochau, K. Preparation and crystal structures of technetium phosphides. *J. Solid. State. Chem.* **44,** 134-140, doi:10.1016/0022-4596(82)90410-8 (1982).
8. Feng, S., Cheng, X., Cheng, X., Yue, J. & Li, J. Theoretical study on electronic, optical properties and hardness of technetium phosphides under high pressure. *Crystals* **7,** 176-185, doi:10.3390/cryst7060176 (2017).
